# Working memory training shows immediate and long-term effects on cognitive performance in children

**DOI:** 10.12688/f1000research.3665.2

**Published:** 2014-11-27

**Authors:** Fiona Pugin, Andreas J. Metz, Madlaina Stauffer, Martin Wolf, Oskar G. Jenni, Reto Huber

**Affiliations:** 1 Child Development Center, University Children’s Hospital Zurich, Zurich, 8032, Switzerland; 2 Zurich Center for Integrative Human Physiology (ZIHP), University of Zurich, Zurich, 8057, Switzerland; 3 Biomedical Optics Research Laboratory, Division of Neonatology, University Hospital Zurich, Zurich, 8091, Switzerland; 4 Division of Neuropsychology, Institute of Psychology, University of Zurich, Zurich, 8050, Switzerland; 5 Children’s Research Center (CRC), University Children’s Hospital Zurich, Zurich, 8032, Switzerland

## Abstract

Working memory is important for mental reasoning and learning processes. Several studies in adults and school-age children have shown performance improvement in cognitive tests after working memory training. Our aim was to examine not only immediate but also long-term effects of intensive working memory training on cognitive performance tests in children. Fourteen healthy male subjects between 10 and 16 years trained a visuospatial n-back task over 3 weeks (30 min daily), while 15 individuals of the same age range served as a passive control group. Significant differences in immediate (after 3 weeks of training) and long-term effects (after 2-6 months) in an auditory n-back task were observed compared to controls (2.5 fold immediate and 4.7 fold long-term increase in the training group compared to the controls). The improvement was more pronounced in subjects who improved their performance during the training. Other cognitive functions (matrices test and Stroop task) did not change when comparing the training group to the control group. We conclude that visuospatial working memory training in children boosts performance in similar memory tasks such as the auditory n-back task. The sustained performance improvement several months after the training supports the effectiveness of the training.

## Introduction

Learning is crucial for adaptation to new situations and is essential for improvements in cognitive functions over time. One important aspect of learning-related improvements in cognitive functions is working memory, which is the ability of simultaneously store and process information held in mind despite irrelevant, potentially interfering stimuli ^1^. It is generally accepted that working memory processes support higher cognitive functions including reasoning ^1^.

Several studies have assessed working memory capacity during childhood and around the age of school entry, at a time when the learning load is large. For example, Alloway *et al.* showed that working memory impairment seems to be disadvantageous for learning abilities in reading and mathematics ^2^. In addition, using the Conners’ Teacher Rating Scale, school teachers have reported high inattentiveness and executive problems in those children with poor working memory ^2^.

Regarding the predictive power of working memory for learning processes, it is not surprising that difficulties in cognitive functions such as reading ^3^ and mathematics ^4^ are associated with working memory skills. For example, Wang *et al.* showed that children between 8 and 10 years with difficulties in single word reading performed worse in simple and complex span tasks compared to controls, two widely used working memory tasks ^3^. In another study, mathematical learning disabilities of school-age children were shown to be associated with low performance in spatial working memory ^4^.

Considering the impact of working memory on a wide range of cognitive functions, the enhancement of working memory is of great interest. Studies in adults showed that working memory may be trained in elderly people of around 80 years ^5^ as well as in young adults in their twenties ^6^. Also in pre-school children, visuospatial working memory training improved untrained verbal working memory performance ^7^. Some of these studies not only showed significant performance increases in the trained working memory tasks, but also in other, untrained working memory tasks ^5^ and functionally more distant tasks such as fluid intelligence tests (transfer effect) ^6^.

In a more recent study involving school-age children of around 9 years, Jaeggi *et al.* also investigated the long-term effects of working memory training ^8^. Immediately after the training, they observed significant higher fluid intelligence in the group with a large training gain compared to the small gain group or a control group. Thus, the larger the training improvement, the greater were the transfer effects. Interestingly, the gain in fluid intelligence remained stable after three months without additional training in the large training gain group ^8^.

The aim of our study was to investigate working memory training and its effects on working memory tasks and fluid intelligence in male subjects between 10 and 16 years. In fact, this age range may be particular susceptible to interventions because many cognitive functions are still developing ^9^. Furthermore, working memory performance has been shown to be linked to attentional control ^10^ and processing speed ^11^. Thus, putative transfer effects on fluid intelligence may not be limited to fluid intelligence, but may also include other cognitive functions. Hence, in addition to working memory and fluid intelligence tasks, we also measured inhibition and interference tasks as well as standardized processing speed. Finally, long-term effects may be indicative for the effectiveness of the training, thus, cognitive testing was repeated not only immediately after the training period but also a few months later.

## Materials and methods

### Subjects

 The participants were recruited through print media and announcements ( *e.g.*, community centers, sport clubs, schools). Inclusion criteria (evaluated by phone screening questionnaires) were: male, age between 10 to 16 years, good general health, right-handedness, no neurological disorder or other disease, no learning disabilities, no smoking or heavy alcohol or caffeine consumption (more than one serving per week). Parents and their children gave written informed consent after explanation of the study methods and aims. Furthermore, ethical clearance was given by the local institutional review board (IRB, KEK-ZH-Nr. 2010-0238/2). In order to assign the subjects to the experimental and control group, we used a stratified randomization with age and cognitive performance in matrices test and letter-number-sequencing (MAT and LNS, see below) as stratification factor.

### Procedure

**Figure 1.**
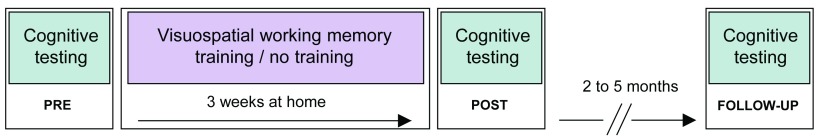
Timeline of the experiment and study procedure. Cognitive testing included two working memory tasks (auditory n-back and letter-number sequencing), a fluid intelligence task [matrix reasoning task, TONI-IV (Test of Non-verbal Intelligence Version IV)], two cognitive control tasks (Stroop and Flanker task), two processing speed tasks (symbol search and digit-symbol substitution task) and a short-term memory task (number-span task). In addition, subjective motivation and concentration were measured on a scale from 1 (minimal) to 10 (maximal).

We assessed the cognitive performance before (PRE) and after 3 weeks (POST) of training. In addition, all subjects were asked to participate in a third session after a minimum of 2 months (FOLLOW-UP = FU, Figure 1). In one subject (control subject, code 36), POST took place 1 week later due to sickness on the planned test date.

Within the 3 weeks between PRE and POST, 14 of the subjects completed an intensive working memory training programme (training group), which consisted of an adaptive visuospatial n-back task ^12^. The control group (N = 15) did not receive any means to perform the training and members were instructed to follow their habitual activities during the three weeks. They did not have access to the training task. However, they were offered to conduct the visuospatial training after the study had been finished.

**Figure 2.**
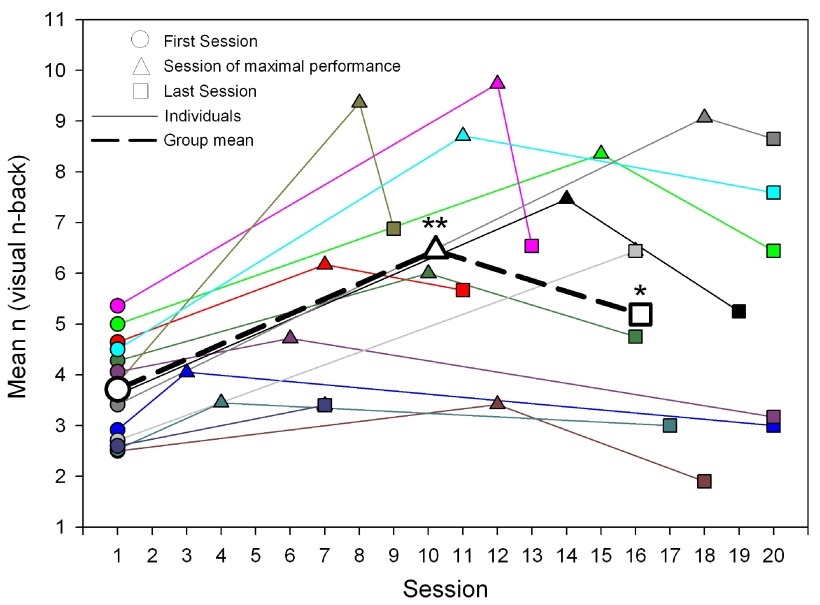
Individual training performance (first session, session of maximal performance, last session). Individual training performance (first session, session of maximal performance, last session) is shown. Each solid line represents the performance of an individual (N = 14) in the visuospatial n-back (VNB) task training mean n of VNB at the first training session (circle), at the session of maximal performance (triangle) and at the last training session (square). The dashed line represents the average performance at the first session, the session of maximal performance and the performance at the last session. Average maximal performance was reached between session 10 and 11 (mean 10.21 ± SEM 1.22) and average performance at the last session was reached between session 16 and 17 (16.14 ± 1.19). ** indicates: performance at session of maximal performance was significantly higher than performance at the first and the last training session. * indicates: performance at the last session was significantly higher than at the first session.

The subjects were unaware of their group affiliation until the end of the first cognitive test session (PRE). The day after PRE testing, each subject from the training group was introduced to the training task by the research assistant. By means of an information sheet, the participants were informed in detail about the task and the setting, and completed a self-motivational control sheet. After this first supervised training session, the participants were able to independently perform the training sessions. Thereafter, the participants were asked to train at home for a maximum of 30 minutes per day over the following 3 weeks. Within these 20 days, they were visited once at home at a planned date by a research assistant (not the cognitive test examiner). During this visit, the working memory training compliance was evaluated by checking the training record stored on the training computer (see Figure 2 for an overview of the number of training session performed by individual subjects). For two subjects, the home visit did not take place due to organizational reasons (control group subjects code 15 and 28). Three weeks after PRE testing, the cognitive testing was repeated (POST) and some months later, the subjects participated in the third test session (FU, range 2 months 22 days to 5 months 6 days). No difference in the timing of FU was observed between the groups (training group: 3 months 21 days ± 6.44 mean ± SEM; control group: 3 months 16 days ± 4.77 mean ± SEM, unpaired t-test).

All participants received a present for taking part in the 3 cognitive test sessions. For participating in the 3 weeks of training, the members of the training group were given a small present of choice.

### Setting

Cognitive performance of all but one subject was assessed by the same examiner. For one subject (code 28), the examiner was different at PRE due to organizational limitations. For each subject, the place of cognitive testing, time of day and week day were kept constant for all three sessions. Due to several reasons ( *e.g*., time limitations or due to participant’s lack of motivation), the number of subjects was unequal for the cognitive tests ( Table 1).

**Table 1.** Mean ± SEM scores for cognitive tests, motivation and concentration. PRE = session 1; POST = session 2, three weeks after session 1; FU = follow-up after 15.59 ± 0.56 weeks (mean ± SEM) after POST. Statistics: mixed ANOVA with factors ‘test session’ (PRE, POST, FU) and ‘group’ (training, control) for each measure. * *p* < 0.05, ^(*)^ < 0.1; ^a^ = age as covariate. Effect size: η ^2^ square; RT = reaction time.

		**TRAINING GROUP**			**CONTROL GROUP**								
*Mean (SEM)* *[Age years]*		12.97 (0.40)			13.23 (0.37)								
		**PRE**	**POST**	**FU**	**PRE**	**POST**	**FU**	**mixed ANOVA**					
	***Measure***	**Mean (SEM)** **[N]**	**Mean (SEM)** **[N]**	**Mean (SEM)** **[N]**	**Mean (SEM)** **[N]**	**Mean (SEM)** **[N]**	**Mean (SEM)** **[N]**	**[group x test** **session]**	***η ^2^***	**[test** **session]**	***η ^2^***	**[group]**	***η ^2^***
*weeks after* *PRE*		-	3	15.92 (0.89)	-	3 (one subject: 4)	15.26 (0.66)						
***Cognitive tests***													
*Working* *memory*	*ANB*	3.46 (0.22) [ 13]	4.92 (0.37) [ 13]	5.17 (0.32) [ 12]	3.71 (0.37) [ 14]	4.29 (0.22) [ 14]	4.07 (0.19) [ 14]	* F(1,23)=7.06 p = 0.003 ^a^	0.235	F=0.48 p=0.60	0.020	^(*)^ F=3.93 p=0.06	0.146
	*LNS*	11.86 (0.57) [ 14]	12.64 (0.85) [ 14]	12.14 (0.64) [ 14]	11.80 (1.07) [ 15]	11.93 (0.56) [ 15]	11.36 (0.44) [ 14]	F(1,25)=0.19 p = 0.78	0.008	F=0.37 p=0.65	0.014	F=0.31 p=0.58	0.012
*Fluid* *Intelligence*	*MAT*	105.29 (3.35) [ 14]	111.29 (2.86) [ 14]	114.07 (2.93) [ 14]	109.80 (3.11) [ 15]	111.13 (2.86) [ 15]	110.64 (3.56) [ 14]	F(1,26)=1.25 p = 0.30	0.05	^(*)^ F=2.93 p = 0.07	0.10	F=0.0 p=0.99	0.000
*Inhibition*	*ST (RT)*	37.87 (4.73) [ 12]	29.76 (3.37) [ 12]	22.56 (2.57) [ 13]	41.99 (5.35) [ 14]	33.16 (4.67) [ 14]	36.15 (6.29) [ 13]	^(*)^ F(1,21)=2.61 p = 0.09 ^a^	0.11	F=2.14 p=0.14	0.09	F=1.843 p=0.19	0.081
*Interference*	*FT (RT)*	1531.88 (404.31) [ 12]	769.84 (265.74) [ 12]	816.81 (168.03) [ 12]	1441.62 (279.08) [ 15]	639.16 (104.77) [ 15]	564.85 (69.08) [ 14]	F(1,23)=0.31 p = 0.65 ^a^	0.01	* F=4.96 p=0.02	0.18	F=0.15 p=0.70	0.007
*Processing* *Speed*	*SST*	11.50 (0.66) [ 14]	12.93 (0.82) [ 14]	13.15 (0.94) [ 13]	11.67 (0.72) [ 15]	13.33 (0.77) [ 15]	13.43 (0.94) [ 14]	F(1,25)=0.05 p = 0.95	0.002	** F=12.08 p=0.00	0.33	F=0.13 p=0.73	0.005
	*DSS*	10.93 (0.53) [ 14]	12.43 (0.76) [ 14]	12.21 (0.61) [ 14]	10.73 (0.83) [ 15]	12.53 (0.90) [ 15]	12.93 (0.95) [ 14]	F(1,26)=1.35 p = 0.27	0.05	** F=17.89 p=0.00	0.41	F=0.01 p=0.91	0.000
*Short-term* *memory*	*NST*	6.08 (0.29) [ 13]	5.92 (0.37) [ 13]	6.31 (0.29) [ 13]	5.6 (0.31) [ 15]	6.07 (0.27) [ 15]	5.64 (0.25) [ 14]	^(*)^ F(1,24)=2.8 p = 0.08 ^a^	0.105	F=0.85 p=0.43	0.034	F=1.48 p=0.24	0.058
***Motivation and concentration***													
*Motivation*	*Scale* *(1 to 10)*	8.17 (0.49) [ 12]	7.42 (0.60) [ 12]	6.67 (0.63) [ 12]	7.93 (0.34) [ 14]	7.14 (0.65) [ 15]	6.77 (0.49) [ 14]	F(1,23)=0.18 p = 0.83	0.008	* F=6.70 p=0.003	0.23	F=0.04 p=0.85	0.002
*Concentration*	*Scale* *(1 to 10)*	7.50 (0.50) [ 12]	6.50 (0.61) [ 12]	6.00 (0.51) [ 12]	7.07 (0.51) [ 14]	6.57 (0.36) [ 15]	6.77 (0.59) [ 14]	F(1,23)=1.03 p = 0.36	0.04	* F=4.09 p=0.03	0.15	F=0.08 p=0.78	0.004

### Intervention

***Visual n-back training [VNBT, computerized version, BrainTwister software,*^12^*].*** One important aspect of working memory training is the adaptation of the difficulty level to the subject’s performance. The working memory load should always be maximal ^13^. For each stimulus of 20 + n consecutive stimuli in a series, the participants had to remember the position of a blue square on a black computer screen and indicate by button-pressing when the square was in the same position as n before. No response was afforded if the square was *not* in the same position as n before. During the test, the participants were supposed to fixate on a cross in the middle of the screen. Per trial, there were 8 possible positions (randomized) of the square (stimulus). Over 20 days, the participants were requested to train for 30 minutes per day. Each of the training sessions started with n = 2 and included several runs (each with a series of 20 + n). After each run, the feedback on performance in percent was displayed (only wrong trial clicks were included in the performance evaluation). The n increased if the performance level was over 90% or decreased with a performance level of 70% or less ^12^.

### Cognitive testing

***Auditory n-back task (ANB, computerized version, BrainTwister software).*** The auditory n-back task ^12^ was based on the same underlying principle as the visuospatial training task, but with computerized spoken letters (C, I, K, Q, W, etc.) instead of visual squares. The duration of the task was restricted to 10 minutes. The maximal n of PRE, POST and FU were used as the dependent variable.

***Letter-number sequencing task (LNS*, *German version of the WISC-IV).*** The letter-number sequencing task was used as an additional task to assess working memory performance ^14^. In this task, the examiner orally presented three times the same length of span (sequence), including a mixture of letters and numbers (e.g., 1 - B - 3 - G - 7). The subject had to remember the span and recite first the numbers in ascending order, then the letters in alphabetical order. As soon as the subject gave an incorrect answer for all three trials for a certain span, the task was finished. The number of the last correct span served as dependent variable (age standardized values according to the normative sample presented in the manual).

***Matrix reasoning task [MAT, Test of Nonverbal Intelligence (TONI IV)].*** As a measure of fluid intelligence, we used the matrix reasoning task TONI-IV ^15^ including two versions, from which the order (A, B, A or B, A, B) was balanced between the groups (age standardized values available). In the task, the participants had to choose the only pattern that completed the matrix presented from a given sample of patterns. The tasks stopped when three of five consecutive trials were not correctly solved or if the maximal trial number was reached (60, none of the subjects reached the maximum).

***Stroop task (ST, paper version).*** For a measure of inhibition, thus inhibiting a response to a distractor stimulus in presence of a target stimulus, we employed a paper version of the Stroop task (one version and no age standardized values available) including three paradigms: 1) reading written color names, 2) naming the colors of lines and 3) naming color-words (incongruent paradigm), where the written color name is different from the ink color of the written word. The participants had to name the ink color of the word as fast as possible, thereby inhibiting to read out the written (semantic) color name. The duration (in seconds) and the errors per paradigm were assessed. As a measure of inhibition, the median time for the incongruent paradigm was used in our study ^16^.

***Flanker task (FT, computerized version).*** As another measure of inhibition, a computerized version of the Flanker task was used. The task was programmed with Presentation 14.8 (Neurobehavioral Systems) according to Stins *et al.* ^17^. In this task, stimulus-response interference is induced by the flanking arrows (distractors) around a middle arrow (target stimulus). Specifically, the participants had to indicate by button pressing (keyboard letter ‘a’ for left and ‘l’ for right) the pointing direction of the middle arrow of the randomly presented trials (condition neutral: < or >, condition congruent: <<<<< or >>>>>, condition incongruent: <<><< or >><>>). The difference between the reaction times for incongruent and congruent trials served as dependent variable.

***Symbol search task and digit symbol substitution task (SST, DSS, German version of the WISC-IV).*** Processing speed was assessed with symbol search and digit symbol substitution task ^14^. These tasks demand for quick and accurate responses, thereby challenging the ability of speed, accuracy and attention. In each trial of the symbol search task, a target symbol was compared with a group of diverse symbols, and the participant indicated with YES or NO whether the chosen symbol was part of the presented group. In the second processing speed task, the DSS ^14^, the participant had to assign a series of numbers to simple geometric symbols, according to a given key. Both tasks had to be solved as quickly as possible. The dependent variable was the number of correct trials within a certain time range (age standardized values).

***Number-span task (NST, KAI).*** The number-span task, assessed with the KAI ^18^, measures short-term memory. In this task, the participants had to repeat a span of numbers that was spoken by the examiner with a regular rhythm of one second. With every correct repetition, the length of the span increased, until the subject gave a false response, resulting in a following span with the same length. With a second error, the task was stopped. The maximally reached span length and the number of errors served as dependent variable (two versions, no age standardized values available).

***Subjective motivation and concentration.*** Before each cognitive test session, the participant was asked to rate his motivation and concentration on a scale ( *1 = not at all* to *10 = highly* motivated or concentrated).

### Analysis and statistics

The training performance was calculated from the individual mean n (n indicates the level of n-back performance, see VNB and ANB above) of each training session. Each session started at n = 2, independent of performance on the previous session. Thus, we decided to consider the first three runs of each session as adaptation runs and excluded them from the analysis. For each training participant, their maximal performance and their respective session were determined over the entire training period. The difference between maximal performance and the last training session (last level) and the level at the first training session (start level) were used as a measure for training gain. The training amount was defined as the number of runs.

Statistical analysis was performed with SPSS software ( PASW Statistics 18). The normal distribution of the cognitive variables was tested with Kolmogorov-Smirnoff test. The effects of the training on cognitive test performance were calculated with a mixed ANOVA, including factor ‘group’ (training, control) and factor ‘test’ (PRE, POST, FU), for each cognitive test. For tests with no age standardized values, age served as a covariate. Unpaired and paired t-tests were calculated for between-group respectively within-group effects. Due to the low number of participants, statistical trends were not considered.

## Results

### Working memory training performance changes

In a first step, we analyzed the visuospatial n-back (VNB) training amount and improvement by comparing performances at the first session with the performances at the last session and with the individual maximal performances ( Figure 2). Participants trained on average for 16.1 ± 1.2 days (mean ± SEM, range: 7 to 20 days). Training performance was significantly increased from the first to the last training session (1.48 ± 0.46 mean ± SEM, range -0.89 to 5.24, paired t-test, *p* < 0.05). Maximal performance was reached on average between session 10 and 11, at 66.9 % ± 7.4 (mean ± SEM, Figure 2) of the individual training time, and was significantly higher than performance at the end of the training (2.74 ± 0.49 mean ± SEM, range 0.66 to 5.65, paired t-test, *p* < 0.001 ( Figure 2).

**Figure 3.**
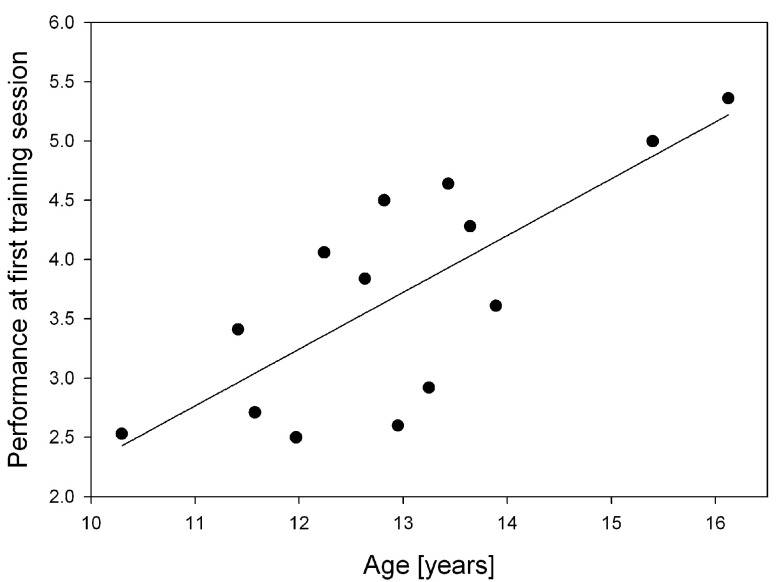
Age at the first training session. Correlation between age (years) and performance at the first session of visuospatial n-back training (Pearson correlation, r = 0.76, *p* < 0.05).

To assess the effect of age on training performance, we performed a correlational analysis. Performance during the first session was positively correlated with age ( Figure 3, Pearson correlation, r = 0.76, *p* < 0.05), that is the older the child, the higher the initial performance. Gain or amount of training, however, was not correlated with age. Initial performance also positively correlated with maximal performance during the training (partial correlation with age as covariate, r = 0.59, *p* < 0.05).

### Effects of working memory training on cognitive performance

**Figure 4.**
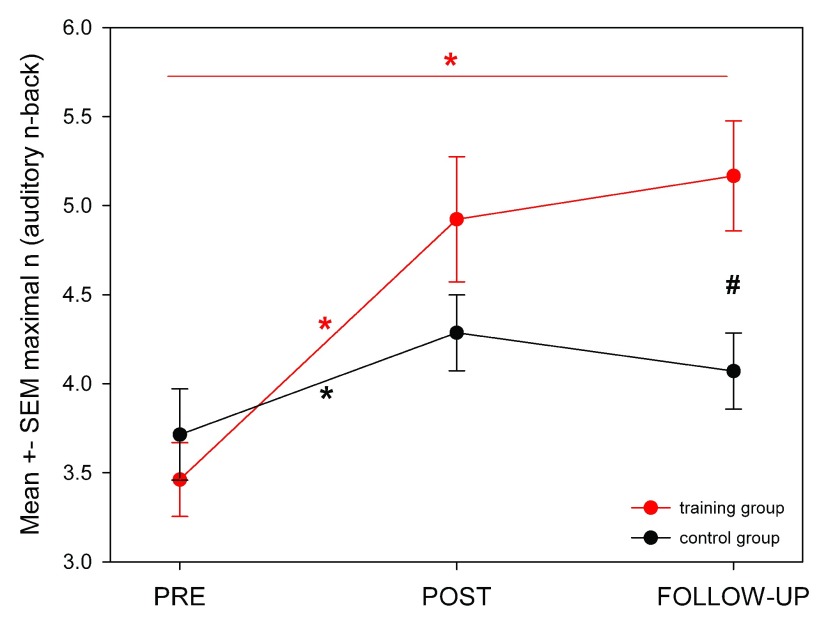
Mean ± SEM of maximal n (auditory n-back, ANB) per group and test session (PRE, POST, FU). The training group showed a significant increase from PRE to POST and to FU. The control group showed a significant increase from PRE to POST, but not FU. * indicates significant changes within group (training (red), control (black); paired t-test, p < 0.05. # indicates significant performance difference at the respective test session (unpaired t-test, p < 0.05)).

In a next step, we analyzed performance in each cognitive test at PRE, POST and FU, comparing the training with the control group ( Table 1). A mixed ANOVA test revealed a significant difference between ‘group’ and ‘test session’ in auditory n-back (ANB) performance ( Table 1). No other test (letter-number sequencing task, number-span task, matrix reasoning task, Stroop task, and Flanker task) showed a significant change. Between-group analysis of performance differences showed significantly higher increases in maximal ANB performance from PRE to POST and to FU in the training compared to controls ( Figure 4). The number of days between PRE and FU (i.e., the time interval between the first and the last session) did not correlate with the improvements in ANB (Pearson correlation).

**Figure 5.**
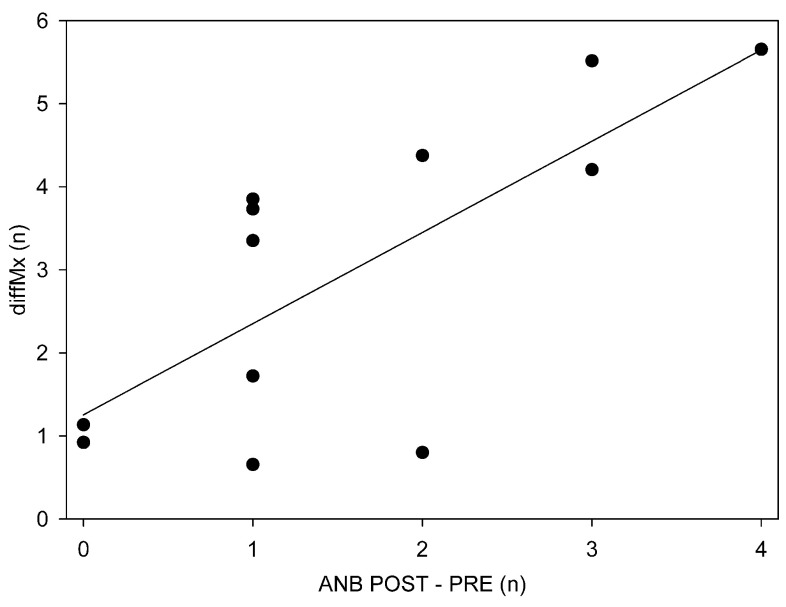
Association between visuospatial training performance increase and auditory n-back (ANB) performance increase. Correlation of training gain (diffMx, difference between Maximal Performance and performance at the first training session) with the change in ANB from PRE to POST (r = 0.76, p < 0.05).

In a following step, we analyzed the improvements in ANB in relation to the training gain and amount. Training gain, measured by the difference in performance between the first session and the session of maximal performance (Pearson correlation, r = 0.76, *p* < 0.05, Figure 5) as well as the difference between the first and the last session (Pearson correlation, r = 0.77, *p* < 0.05), correlated positively with the improvements from PRE to POST in ANB. No correlation between ANB increase and training amount was found.

### Individual training performance

**Figure 6.**
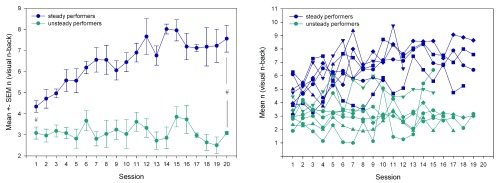
Visuospatial training performance for the steady and unsteady performers. Steady performers: individuals with a significant positive correlation between mean training performance (mean n) per session and the training session. Unsteady performers: no positive correlation. Left: mean ± SEM of n [visuospatial training task (VNB)] over each training session per group. Right: individual mean n (VNB) per training session. Blue: steady performer group. Green: unsteady performer group. # indicates significant performance differences between steady and unsteady performers at the first and the last session (unpaired t-test, p < 0.05).

For assessing how training performance gain may influence the gain in auditory n-back (ANB), we grouped the subjects according to their training performance, by correlating the training performance over time with the session number ( Figure 6). In seven of the 14 training subjects, training days positively correlated with daily performance, indicating a steady increase of training performance over the entire training period (steady performer group). In the remaining subjects, the training performance was not stable and/or decreased (unsteady performer group).

In addition to the steady increase, the steady performers started on a significantly higher level ( Figure 6) and showed a larger increase in training performance from the first session to the session of maximal performance and to the performance at the last session (unpaired t-test, *p* < 0.05).

**Figure 7.**
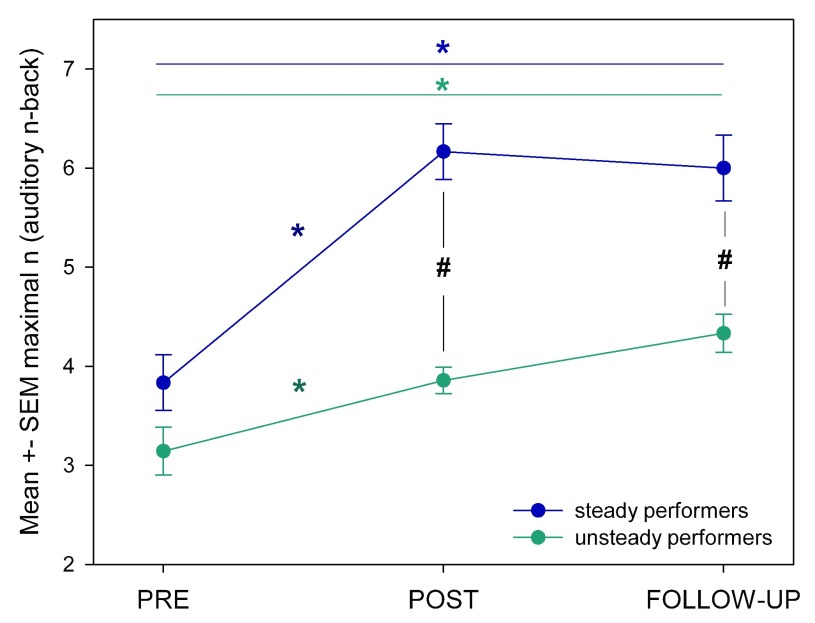
Mean ± SEM in auditory n-back (ANB) for the steady and unsteady training performers. * indicates significant changes within groups [steady performers (blue), unsteady performers (green)]. # indicates p < 0.05 (unpaired t-test between sessions [PRE, POST, FU]).

When we then compared ANB performance between these two groups, we found that the steady performers showed a significant higher increase from PRE to POST and higher increase in maximal ANB performance at POST and FU compared to the unsteady performers ( Figure 7).

## Discussion

During three weeks of visuospatial memory training, the participants in this study showed a significant performance increase in auditory working memory compared to passive controls. The better performance remained high after some months. The performance improvement correlated with the training quality, that is the performance level, rather than with the amount of training. The cognitive control, measured with the Stroop and the Flanker task, was not associated with the performance increase. No significant transfer effects on fluid intelligence were observed.

### Visual n-back training improves related working memory task performance

The strongest effect of VNB training was found on ANB, a working memory task closely related to the one trained for by our participants. Importantly, not the amount of training but the training gain correlated positively with the immediate and long-term increase in the auditory working memory task: the higher the increase in ANB from PRE to POST and to FU, the higher the training gain. Our approach based on correlating the individual training sessions with the training performance at each session supports the finding that training performance rather than the amount of training is crucial for the increase in ANB. The correlations show that the participants with a steady increase of their training performance had larger gains in training performance as well as in immediate and long-term ANB performances. However, the influencing factors for a steady performance increase are not known. Further studies may show if such differences in training performance may be diminished by enhancing the individual’s motivation or by an improved guidance and control through the daily training sessions.

In agreement with our results, few other studies reported long-term effects of working memory training on the working memory performance ^19, 20^. In young and old adults, n-back task performance was maintained three months after spatial working memory training ^20^. Dahlin *et al.* found stable improvements even eighteen months after the training ^19^. One explanation for such long-term effects in auditory working memory after training might be that training leads to more efficient use of working memory in daily life not only during the sessions, but also afterwards. A relationship between the efficient use of working memory and long term benefits for cognitive performance is supported by the observation that working memory tasks such as the digit span task and the visual-spatial working memory task were shown to predict mathematical and reading skills achieved in the first school years ^21^. The transfer from test setting to daily life would be a convincing rationale to apply working memory training as a therapeutic tool. Indeed, in children with poor working memory performance, Holmes *et al*. found significant performance increases in mathematics 6 months after an intensive working memory training ^22^. Thus, long-term measures of cognitive performance may indicate a certain effectiveness of cognitive training. If intensive working memory training is effective, a more efficient use of working memory would transfer into daily life, for example resulting in higher grades at school.

The idea that the observed improvements in visuospatial and auditory working memory would have occurred due to a use-dependent general increase in working memory capacity, may still be questioned by our finding that the letter-number sequencing, a typical task assessing working memory capacity, was not affected by training. This finding is in line with other studies comparing working memory capacity ( *e.g.*, by digit span tasks) and n-back tasks ^6, 23^. As Kane *et al.* suggested ^23^, n-back tasks may ‘not reflect primarily a single construct’ because ‘complex span tasks typically demand serial recall’, whereas ‘n-back tasks typically demand recognition’. Thus, serial recall and recognition do not correlate. This observation may indicate that the auditory and visuospatial tasks are actually too similar to draw conclusions about general effects on working memory *per se*.

### Working memory training does not significantly improve fluid intelligence

We found no significant effects of the training on non-verbal matrices test when comparing the training group to the control group. Also Jaeggi *et al.,* when comparing fluid intelligence test performance between the experimental and control group and between three test sessions, did not find any effect of visuospatial working memory training on fluid intelligence ^8^. Only after grouping the participants according to their training gain in a large and small training gain group (median split) some differences in transfer effects were observed: immediate transfer effects and, to lesser extent, long-term transfer effects were significantly higher in the large training gain group compared to the low training gain and control group. When we performed the same median split in our training group, we were not able to find any statistically significant transfer effects. However, several aspects, such as the training duration and the attractiveness of the training, render it difficult to directly compare the two studies. After all, in a study with adult participants, the transfer effects of working memory training on fluid intelligence could not be replicated ^24^. Furthermore, in a recently presented meta-analysis that investigated the effects of working memory training on cognitive tasks, Melby-Lervåg and Hulme revealed that working memory training indeed improves the working memory capacity immediately after the training. The training, however, does not lead to transfer effects on fluid intelligence when focusing on studies with control groups and randomization ^25^. The major limitations of this study are the low number of subjects and the lack of an active control group. However, despite these limitations, our negative finding concerning transfer effects on fluid intelligence is congruent with the current literature. In fact, the passive control group had a very low bar to pass, and still no far-transfer effects were found. Thus, this finding may reflect strong evidence against far transfer effects.

### Working memory training does not affect processing speed and is not related to cognitive control

As proposed by Diamond and Lee ^26^, working memory training does not improve inhibition or processing speed. Our data are in line with this notion, since we did not find any effects of the training on the processing speed tasks and the Stroop and Flanker test. The training and the control groups increased processing speed over the three sessions similarly. As shown by Conway *et al.*, digit-symbol tasks may rather reflect short-term memory than working memory ^27^. However, our short-term memory task was not affected by working memory training. Thus, we conclude that processing speed, used for measuring digit-symbol and symbol search tasks, are unaffected by working memory training and/or practice effects may be stronger.

Our data did not indicate that cognitive control, measured with the Stroop and Flanker task, is associated with the training gain and effects on auditory working memory. Although our results do not corroborate the theory of attentional control being a crucial factor for working memory ^28^, we do not question the necessity of cognitive control for reasoning. Again, with our data sample of limited size and large inter-individual variability we may not have been able to capture factors that influence working memory performance gains or even changes in cognitive functions due to training.

We conclude from these results that the performance increase in a visuospatial working memory task is beneficial for auditory working memory in children. The dominant long-term effects underline the importance of assessing performance not only right after the cognitive training, but also several months later. Regarding the importance of working memory for other cognitive functions, studies comparing populations of different developmental stage or of low and high working memory capacity are needed.

## Data availability

The ethical approval granted to the authors by the IRB does not allow the publication of the raw data online. If readers would like to re-analyze the data set (for different purposes), additional ethical approval (on a individual user and purpose basis) will be required. The authors would be happy to support additional ethical approval applications from researchers for access to this data set.

**Consent**

Informed written consent was provided by the children participating in the study and their parents.
