# Supplementary material for: Working memory training shows immediate and long-term effects on cognitive performance in children
Source: F1000Res. 2014 Nov 27;3:82. Originally published 2014 Apr 2. [Version 2] doi: 10.12688/f1000research.3665.2 (PMC4309169; doi:10.12688/f1000research.3665.2)
Supplement: Peer review history

# Peer review: Referee response for version 2

**DOI:** 10.5256/f1000research.6175.r6833

**Published:** 2014-12-19

**Authors:** Silvia Bunge

**Affiliations:** 1 Department of Psychology, University of California Berkeley, Berkeley, CA, USA

The authors report their results clearly, and articulate the limitations of their study. Given the relatively low N, they would do well to temper the general statements made in the subheadings of the Discussion section (e.g., "Working memory training does not affect processing speed and is not related to cognitive control").

I have read this submission. I believe that I have an appropriate level of expertise to confirm that it is of an acceptable scientific standard.

# Response

**Published:** 2014-12-22

**Authors:** Fiona Pugin

**Affiliations:** University Children's Hospital Zurich, Switzerland

Dear Professor Bunge

Thank you for calling our attention to the inconsistency between the discussion text and its subheadings. We mitigated the statement of the subheadings by using the word ‘ *may*’ respectively ‘ *seems to*’.

**Visual n-back training *may *improve related working memory task performance**

**Working memory training *seems *not *to* significantly improve fluid intelligence**

**Working memory training *seems *not *to* affect processing speed and *seems* not *tobe* related to cognitive control**

# Peer review: Referee response for version 2

**DOI:** 10.5256/f1000research.6175.r7053

**Published:** 2014-12-17

**Authors:** Eddy van der Zee

**Affiliations:** 1 Centre for Behaviour and Neurosciences, University of Groningen, Groningen, Netherlands

I am satisfied with the way the authors addressed my questions and concerns. I have no further issues that need to be dealt with.

I have read this submission. I believe that I have an appropriate level of expertise to confirm that it is of an acceptable scientific standard.

# Peer review: Referee response for version 1

**DOI:** 10.5256/f1000research.3929.r4725

**Published:** 2014-09-02

**Authors:** Eddy van der Zee

**Affiliations:** 1 Centre for Behaviour and Neurosciences, University of Groningen, Groningen, Netherlands

This work shows the immediate and long-term effects of working memory training on cognitive performance of children (age 10-16). It is an interesting finding that 30 min of daily visuo-spatial working memory training can have sustained improvement in an auditory n-back task. Although the results seem to be sound despite the relative low group sizes and the lack of an active control group, some issues need further attention:

1. It is not made clear enough what the passive control group is doing (or was instructed NOT to do) and particularly how this was controlled for.
2. Although Table 1 is of help, the memory training compliance was said to be evaluated by a research assistant. Can more information on this evaluation be provided to convince the reader that it is likely that the compliance of the children was indeed as planned?
3. Can more be said about the cause of the individual differences in training performance (the steady and unsteady performer groups)? Is it anticipated that individuals of the unsteady performer group, if their performance would have been steady (for example by more strict control or guidance), would gain as much as individuals of the steady performer group?
4. In the end of the abstract, spatial working memory should be visuo-spatial working memory.
5. Given the age range of the kids and the low number of children over 14 (n=2?) in this study it seems not fully justified to speak about adolescents if it comes to the effects of working memory training.

I have read this submission. I believe that I have an appropriate level of expertise to confirm that it is of an acceptable scientific standard, however I have significant reservations, as outlined above.

# Response

**Published:** 2014-11-14

**Authors:** Fiona Pugin

**Affiliations:** University Children's Hospital Zurich, Switzerland

Dear Professor van der Zee,

We would like to thank you for your helpful comments on the manuscript. We have addressed all of them in our point by point reply below.

1. It is not made clear enough what the passive control group is doing (or was instructed NOT to do) and particularly how this was controlled for.
**The subjects of the control group did not receive the means to train (laptop computer with training software installed). They were instructed to follow their habitual daily routines during the 3 weeks (during which the training group subjects conducted their training).**
**We added this information in the ‘Materials and methods / Procedure‘ section:**
***“The control group (N = 15) did not receive any means to perform the training and were instructed to follow their habitual activities during the three weeks. They did not have access to the training task. However, they were offered to conduct the visuospatial training after the study had been finished.”***
2. Although Table 1 is of help, the memory training compliance was said to be evaluated by a research assistant. Can more information on this evaluation be provided to convince the reader that it is likely that the compliance of the children was indeed as planned?
**The training software (‘Brain Twister’) stores the training record of the individual subjects. Figure 2 displays the training compliance of the subjects by indicating the number of training sessions which were conducted by the individuals during the training period of three weeks (x-axis). We extended the relevant sentence as follows:**
***“During this visit, the working memory training compliance was evaluated by checking the training record stored on the training computer (see Figure 2 for an overview of the number of training session performed by individual subjects).”***
3. Can more be said about the cause of the individual differences in training performance (the steady and unsteady performer groups)? Is it anticipated that individuals of the unsteady performer group, if their performance would have been steady (for example by more strict control or guidance), would gain as much as individuals of the steady performer group?
**This is an interesting question; however, we have no data to inquire more about the cause of performance differences. To answer the question if a stricter and continuous control of the training compliance would have an influence on the steadiness of the performance, further studies are required. We added the following to the ‘Discussion‘ section:**
***“However, the influencing factors for a steady performance increase are not known. Further studies may show if such differences in training performance may be diminished by enhancing the individual’s motivation or by an improved guidance and control through the daily training sessions.”***
4. In the end of the abstract, spatial working memory should be visuo-spatial working memory.
**This issue was corrected.**
5. Given the age range of the kids and the low number of children over 14 (n=2?) in this study it seems not fully justified to speak about adolescents if it comes to the effects of working memory training.
**We appreciate this comment (there are two subjects over 14 years in the training group and four subjects in the control group). Accordingly, we changed the wording in the title as well as in the text by limiting our statements such that they refer to children only.**
**New Title: *“Working memory training shows immediate and long-term effects on cognitive performance in children”***
**Changes in the abstract:**
***“Our aim was to examine not only immediate but also long-term effects of intensive working memory training on cognitive performance tests in children.”***
***“We conclude that visuospatial working memory training in children boosts performance in similar memory tasks such as the auditory n-back task.”***
**Change in the discussion:**
***“We conclude from these results that the performance increase in a visuospatial working memory task is beneficial for auditory working memory in children”. ***

# Peer review: Referee response for version 1

**DOI:** 10.5256/f1000research.3929.r5128

**Published:** 2014-07-02

**Authors:** Silvia Bunge

**Affiliations:** 1 Department of Psychology, University of California Berkeley, Berkeley, CA, USA

This study examines the immediate and long-term cognitive effects of a 20-day visuospatial working memory training program in a small group of 10-16-year-old boys (N=14), in comparison with a passive control group (N=15). The intervention group showed improvements in visuospatial n-back task (training task) and an auditory n-back task (transfer task) that were significantly greater than those observed for the passive control group. Gains in the auditory n-back task were sustained at a three-month follow-up assessment. Measures of fluid intelligence, inhibition, processing speed, and other working memory tasks (letter-number sequencing task) did not show transfer effects at any time point, as judged by significance testing. The authors show differences in the degree of improvement on the transfer task as a function of training gains on the n-back task (steady vs. unsteady performers). This study adds to a growing body of work establishing the boundary conditions of cognitive training effects. Limitations of this study include a small sample size, the absence of an active control group, and the failure to report on the motivation ratings gathered at each session, which could help to clarify differences between the steady and unsteady performers.

I have read this submission. I believe that I have an appropriate level of expertise to confirm that it is of an acceptable scientific standard, however I have significant reservations, as outlined above.

# Response

**Published:** 2014-11-14

**Authors:** Fiona Pugin

**Affiliations:** University Children's Hospital Zurich, Switzerland

Dear Professor Bunge, we would like to thank you for your helpful comments on the manuscript. We have addressed all of them in our point by point reply below.

1. Limitations of this study include a small sample size, the absence of an active control group,
**We changed the wording in the ‘Discussion‘ section:**
*****“The major limitations of this study are the low number of subjects and the lack of an active control group. However, despite these limitations, our negative finding is […]”*****
2. and the failure to report on the motivation ratings gathered at each session, which could help to clarify differences between the steady and unsteady performers.
**This is a similar question as asked by reviewer Prof. van der Zee: see for point 3 in the response to Prof. van der Zee.**
